# Effects of thiram exposure on liver metabolism of chickens

**DOI:** 10.3389/fvets.2023.1139815

**Published:** 2023-02-28

**Authors:** Meng Wang, Lei Wang, Sana Shabbir, Dongliang Zhou, Muhammad Akbar Shahid, Houqiang Luo, Huixia Li, Ziwei Li, Xingya Sun, Chunqin Wu, Yan Zhao

**Affiliations:** ^1^College of Animal Science, Wenzhou Vocational College of Science and Technology, Wenzhou, China; ^2^College of Veterinary Medicine, Huazhong Agricultural University, Wuhan, China; ^3^Punjab Health Department, Punjab, Pakistan; ^4^Department of Pathobiology, Faculty of Veterinary Sciences, Bahauddin Zakariya University, Multan, Pakistan

**Keywords:** thiram, liver, metabolism, chicken, pesticides

## Abstract

Pesticides are widely used to control crop diseases, which have made an important contribution to the increase of global crop production. However, a considerable part of pesticides may remain in plants, posing a huge threat to animal safety. Thiram is a common pesticide and has been proven that its residues in the feed can affect the growth performance, bone formation, and intestinal health of chickens. However, there are few studies on the liver metabolism of chickens exposed to thiram. Here, the present study was conducted to investigate the effect of thiram exposure on liver metabolism of chickens. Metabolomics analysis shows that 62 metabolites were down-regulated (ginsenoside F5, arbekacin, coproporphyrinogen III, 3-keto Fusidic acid, marmesin, isofumonisin B1, 3-Hydroxyquinine, melleolide B, naphazoline, marmesin, dibenzyl ether, etc.) and 35 metabolites were up-regulated (tetrabromodiphenyl ethers, deoxycholic acid glycine conjugate, L-Palmitoylcarnitine, austalide K, hericene B, pentadecanoylcarnitine, glyceryl palmitostearate, quinestrol, 7-Ketocholesterol, tetrabromodiphenyl ethers, etc.) in thiram-induced chickens, mainly involved in the metabolic pathways including glycosylphosphatidylinositol (GPI)-anchor biosynthesis, porphyrin and chlorophyll metabolism, glycerophospholipid metabolism, primary bile acid biosynthesis and steroid hormone biosynthesis. Taken together, this research showed that thiram exposure significantly altered hepatic metabolism in chickens. Moreover, this study also provided a basis for regulating the use and disposal of thiram to ensure environmental quality and poultry health.

## Introduction

Increasing evidence indicated that pesticides play a vital role in agricultural production. Statistical analysis indicated that China is the main consumer of pesticides, using 1.8 million tons per year, followed by the America (1–3). At present, pesticides have been listed as priority pollutants by the United Nations Environment Protection Agency (UNEP) (4, 5). Although, the use of pesticides has effectively increased crop yield and reduced disease. However, the extensive use of pesticides will also cause serious environmental pollution, posing a serious threat to food security and animal health (6). In addition, some pesticides may remain in plants and be introduced into nearby waters after rainfall, endangering the health of aquatic animals and causing drinking water safety problems (7–16). Moreover, aerial spray of pesticides may cause the pollution of nearby or distant areas through transboundary movement (17). It is worth noting that humans and animals may also ingest plants containing pesticides through the food chain, seriously endangering public health and human security (18–21).

Poultry including chickens, ducks and geese are the largest livestock species. These species developed rapidly in the past few decades, effectively solving the problem of protein shortage. Among the above-mentioned poultry, chickens are widely farmed because of their fast growth and low price (22, 23). Consequently, any factors that endanger chickens should be given enough attention. However, chickens are likely to be exposed to feed containing pesticide residues (24–27). Previous studies have indicated that most pesticides could accumulate in multiple tissues and inhabit the exposed organisms from few months to several years, thus even very low concentration is also harmful to health (5, 28, 29). Liver is the vital metabolic and alexipharmic organ in the animal and humans, which is considered as one of the primary target organs for various hazardous substances such as pesticides and heavy metal (30–32). Therefore, the intake of feed containing pesticide residues will inevitably affect the liver of broilers.

Thiram is one of the common pesticides, mainly used to increase crop yield and reduce disease (33, 34). However, the abuse of thiram not only cause pesticide residues, but also pose a serious threat to the safety of humans and animals (35–37). Previous studies have shown that thiram exposure causes abnormal bone development and reduced growth performance in chickens (38–40). In addition, thiram exposure has been demonstrated to cause intestinal flora imbalance and liver histopathology injuries in chickens (18, 26). However, studies regarding the influences of thiram exposure on liver metabolism in chickens remain scarce. Taking advantage of this gap, we explored the effect of thiram exposure on liver metabolism in chickens.

## Materials and methods

### Animal experiments and sample acquisition

A group of 60 one-day-old healthy Arbor Acres chickens were purchased from a commercial hatchery and maintained under the standard ambient temperature, sanitary condition and illumination as previously described. Prior to the experiment, all the subjects were performed physical examinations to avoid deformity and other congenital diseases. After acclimatization for 3 days, an equal number of chickens (n = 30) regardless of sex were divided into control and thiram-treated groups. Throughout the trial, the control chickens were provided sufficient feed and water. Moreover, the chickens in thiram-treated group received same diet as controls but supplemented with thiram (50 mg/kg) purchased from Macklin Biochemical Co., Ltd. (Shanghai, China) in feed as suggested by previous research from days 3–7 (39). All chickens were euthanized and liver tissue was collected on days 18 of the experimental study. The achieved samples were snap-frozen utilizing liquid nitrogen and stored at −80°C for further study.

### Sample preparation

The metabolomic procedure was conducted based on the previous protocols with minor improvements (41, 42). Briefly, the acquired liver samples (~100 mg) were triturated in methanol and then centrifuged for 15 min at 14,000×*g*. The supernatant of mixture was collected and stored in Eppendorf tubes for 10 min. Subsequently, the deionized water (400 μl) was added to the obtained supernatant and kept at −80°C for further study. The extract (100 μl) of each sample was mixed for preparing quality control (QC) sample and QC samples were performed testing between every five samples. The 0.22 μm membranes were applied to filter the supernatant and then the filtered supernatant was performed UPLC-QTOF/MS (Waters, USA) analysis. The condition of UPLC was determined as described previously (42). Moreover, the reagents used in this study were HPLC grade.

### Differential metabolite analysis

The original mass spectrometry was subjected to process using Marker View 1.1 (AB SCIEX, USA). Subsequently, PCA and PLS-DA were performed by importing metabolomics data into SIMCA (version 14.1, Umetrics, Sweden). The determination of differential metabolites was based on the variable weight value (VIP) and *p*-value obtained from the OPLS-DA model. To obtain pathways involved in differential metabolites, MetaboAnalyst and KEGG database (https://www.kegg.jp/kegg/pathway.html) was used to perform cluster analysis and metabolic pathway annotation of differential metabolites.

## Results

### Thiram exposure disrupts liver metabolism

**Figure 1:**
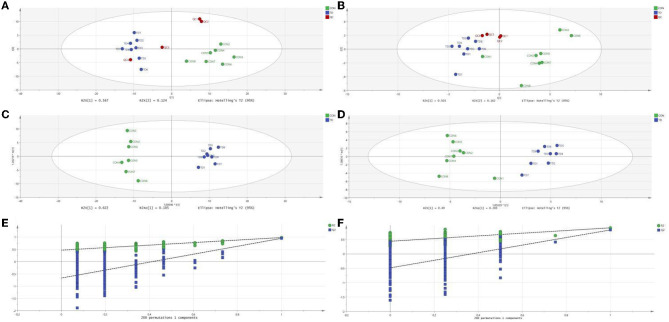
Thiram exposure altered liver metabolism. **(A, B)** PCA score plots based on positive-ion mode and negative-ion mode, respectively. **(C, D)** OPLS-DA plot based on positive-ion mode and negative-ion mode, respectively. **(E, F)** Permutation tests based on positive-ion mode and negative-ion mode, respectively.

The plots of PCA analysis showed that the samples in the thiram-exposed group were clustered closely and separated from the control group, indicating that thiram exposure significant changes in liver metabolome (Figures 1A, B). To further reveal the alterations of liver metabolome during thiram exposure, OPLS-DA score plots was applied for pattern discriminant analysis. Results indicated that there was a clear separation between both groups and no fitting occur (Figures 1C–F).

### Identification of metabolites associated with thiram exposure

**Figure 2:**
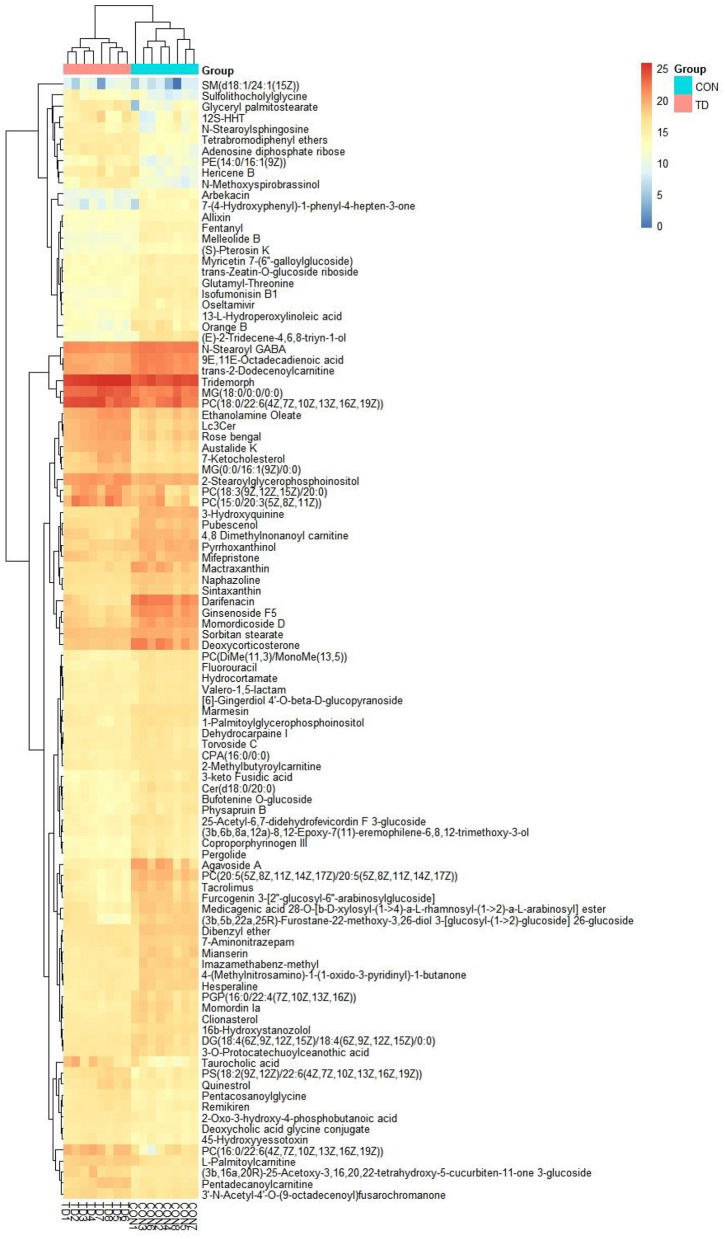
Heatmap revealed the differential metabolites in liver exposed to thiram. The color in the heatmap indicates the normalized relative abundance of each metabolite.

The differential metabolites were recognized based on the criterion of VIP > 1, *P* < 0.05. Results indicated that a total of 97 differential metabolites were detected between both groups (Table 1). Among significantly different metabolites, 62 metabolites (ginsenoside F5, arbekacin, coproporphyrinogen III, 3-keto Fusidic acid, marmesin, etc.) were down-regulated, whereas 35 metabolites (L-Palmitoylcarnitine, quinestrol, 7-Ketocholesterol, tetrabromodiphenyl ethers, etc.) were up-regulated in thiram-induced chickens. Moreover, the alternations of metabolites also could be observed in the heatmap (Figure 2).

**Table 1:** Statistical analysis of differential metabolites between thiram-exposed and control groups.

**Metabolites**	**Fold change**	**VIP**	**Trend**
Isofumonisin B1	9.33	1.21	Down
3-Hydroxyquinine	5.86	1.20	Down
16b-Hydroxystanozolol	2.87	1.19	Down
9E,11E-Octadecadienoic acid	3.70	1.19	Down
Melleolide B	6.80	1.19	Down
Naphazoline	2.64	1.19	Down
Marmesin	3.32	1.19	Down
Dibenzyl ether	3.72	1.19	Down
Ginsenoside F5	11.28	1.19	Down
Imazamethabenz-methyl	4.93	1.18	Down
Darifenacin	30.79	1.18	Down
7-Aminonitrazepam	2.87	1.18	Down
Cer(d18:0/20:0)	7.18	1.17	Down
CPA(16:0/0:0)	2.67	1.17	Down
Momordicoside D	4.69	1.17	Down
Sintaxanthin	2.80	1.17	Down
Valero-1,5-lactam	2.13	1.17	Down
Tacrolimus	14.95	1.17	Down
Tetrabromodiphenyl ethers	3.69	1.17	Up
Pubescenol	3.28	1.16	Down
Sorbitan stearate	2.13	1.16	Down
[6]-Gingerdiol 4'-O-beta-D-glucopyranoside	2.68	1.16	Down
Allixin	2.19	1.16	Down
Fentanyl	3.74	1.16	Down
trans-2-Dodecenoylcarnitine	2.92	1.16	Down
PC(DiMe(11,3)/MonoMe(13,5))	3.65	1.16	Down
Pyrrhoxanthinol	3.14	1.16	Down
Bufotenine O-glucoside	3.60	1.15	Down
Hesperaline	4.72	1.15	Down
Momordin Ia	5.63	1.15	Down
Fluorouracil	3.66	1.15	Down
Deoxycholic acid glycine conjugate	2.07	1.15	Up
Coproporphyrinogen III	6.16	1.14	Down
Furcogenin 3-[2″-glucosyl-6″-arabinosylglucoside]	9.88	1.14	Down
Clionasterol	3.77	1.14	Down
L-Palmitoylcarnitine	2.70	1.14	Up
trans-Zeatin-O-glucoside riboside	2.40	1.14	Down
Myricetin 7-(6″-galloylglucoside)	2.80	1.14	Down
2-Methylbutyroylcarnitine	2.06	1.13	Down
Austalide K	4.74	1.13	Up
Glutamyl-Threonine	3.68	1.13	Down
25-Acetyl-6,7-didehydrofevicordin F 3-glucoside	5.65	1.12	Down
Adenosine diphosphate ribose	5.37	1.12	Up
N-Stearoyl GABA	2.04	1.12	Down
Lc3Cer	4.74	1.12	Up
(S)-Pterosin K	3.00	1.12	Down
Pentacosanoylglycine	3.96	1.11	Up
Ethanolamine Oleate	4.42	1.11	Up
Agavoside A	26.93	1.10	Down
Hericene B	3.19	1.10	Up
Pentadecanoylcarnitine	3.82	1.10	Up
Glyceryl palmitostearate	10.38	1.10	Up
Oseltamivir	4.65	1.10	Down
MG(18:0/0:0/0:0)	2.80	1.09	Up
Physapruin B	3.92	1.09	Down
N-Methoxyspirobrassinol	10.02	1.09	Up
Mactraxanthin	7.83	1.09	Down
Deoxycorticosterone	8.73	1.08	Down
2-Oxo-3-hydroxy-4-phosphobutanoic acid	2.23	1.08	Up
4,8 Dimethylnonanoyl carnitine	3.36	1.08	Down
Tridemorph	2.56	1.08	Up
PE([14:0/16:1(9Z)))]	3.31	1.07	Up
1-Palmitoylglycerophosphoinositol	3.02	1.07	Down
45-Hydroxyyessotoxin	2.37	1.07	Up
PS([18:2(9Z,12Z)/22:6(4Z,7Z,10Z,13Z,16Z,19Z)))]	3.55	1.07	Up
Rose bengal	3.62	1.06	Up
Remikiren	2.58	1.06	Up
Hydrocortamate	2.05	1.06	Down
DG[18:4(6Z,9Z,12Z,15Z)/18:4(6Z,9Z,12Z,15Z)/0:0]	2.53	1.06	Down
Dehydrocarpaine I	2.28	1.06	Down
2-Stearoylglycerophosphoinositol	2.50	1.05	Up
Torvoside C	2.06	1.04	Down
Orange B	10.70	1.04	Down
7-Ketocholesterol	5.39	1.04	Up
MG(0:0/16:1(9Z)/0:0)	2.48	1.04	Up
Pergolide	8.78	1.03	Down
PGP[16:0/22:4(7Z,10Z, 13Z,16Z)]	3.33	1.03	Down
3-O-Protocatechuoylceanothic acid	3.37	1.02	Down
3'-N-Acetyl-4'-O-(9-octadecenoyl)fusarochromanone	2.12	1.01	Up
PC[18:0/22:6(4Z,7Z,10Z, 13Z,16Z,19Z)]	3.38	1.01	Up
Mifepristone	2.14	1.00	Down
Quinestrol	3.48	1.00	Up
(E)-2-Tridecene-4,6,8-triyn-1-ol	26.33	1.48	Down
PC[16:0/22:6(4Z,7Z,10Z,13Z, 16Z,19Z)]	6.05	1.28	Up
Arbekacin	8.07	1.25	Down
7-(4-Hydroxyphenyl)-1-phenyl-4-hepten-3-one	11.68	1.24	Down
Sulfolithocholylglycine	4.91	1.23	Up
Taurocholic acid	7.22	1.23	Up
12S-HHT	7.82	1.16	Up
SM[d18:1/24:1(15Z)]	18.06	1.16	Up
N-Stearoylsphingosine	2.96	1.07	Up
PC(18:3(9Z,12Z,15Z)/20:0)	4.09	1.07	Up
Mifepristone	14.97	1.05	Up
PC[15:0/20:3(5Z,8Z,11Z)]	4.33	1.04	Up
Mianserin	4.37	1.04	Down
3-keto Fusidic acid	4.16	1.01	Down
13-L-Hydroperoxylinoleic acid	4.59	1.00	Down

### Metabolic pathway analysis

**Figure 3:**
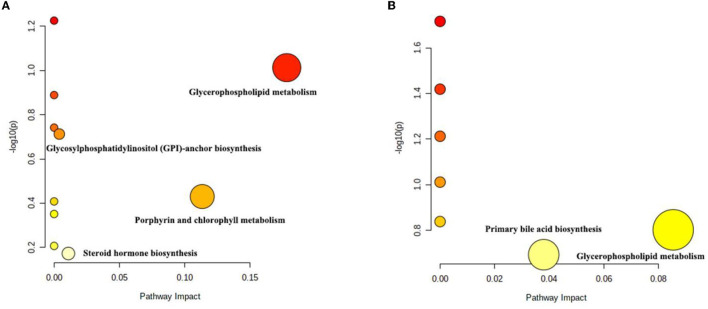
Differential metabolic pathway analysis based on the positive-ion mode **(A)** and the negative-ion mode **(B)**. Each circle represents a metabolic pathway.

**Figure 4:**
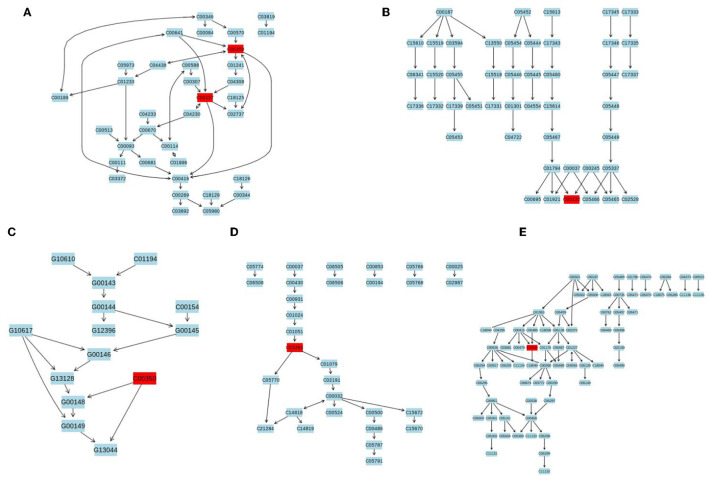
The representative schematic diagram of liver metabolic exposed to thiram. **(A)** Glycerophospholipid metabolism. **(B)** Primary bile acid biosynthesis. **(C)** Glycosylphosphatidylinositol (GPI)-anchor biosynthesis. **(D)** Porphyrin and chlorophyll metabolism. **(E)** Steroid hormone biosynthesis. The red boxes represent the differential metabolites associated with thiram exposure.

The differential metabolites were subjected to pathway analysis by utilizing MetaboAnalyst 4.0 and results indicated that 13 metabolic pathways (linoleic acid metabolism, glycerophospholipid metabolism, taurine and hypotaurine metabolism, vitamin B6 metabolism, alpha-Linolenic acid metabolism, glycosylphosphatidylinositol (GPI)-anchor biosynthesis, sphingolipid metabolism, porphyrin and chlorophyll metabolism, arachidonic acid metabolism, fatty acid degradation, primary bile acid biosynthesis, purine metabolism and steroid hormone biosynthesis) involved in hepatotoxicity induced by thiram (Figure 3). Among above-mentioned differential pathways, 5 pathways with highest pathway impact value were the glycosylphosphatidylinositol (GPI)-anchor biosynthesis, porphyrin and chlorophyll metabolism, glycerophospholipid metabolism, primary bile acid biosynthesis and steroid hormone biosynthesis. The metabolic diagram in the intestine is shown in Figure 4.

## Discussion

Thiram is widely used in agricultural production and is likely to accumulate in plants (43–45). Some plant-sourced feeds that accumulate pesticides are likely to enter poultry farming through the food chain, posing a serious threat to the health of poultry (18, 38, 39). At present, the harm of thiram exposure to various species such as mice, chickens and fish has been widely confirmed. For instance, thiram has been shown to dramatically affect the respiratory tract, central nervous system, stimulate skin and restrain the formation of white blood cells (34, 46, 47). Furthermore, some studies have also demonstrated the role of thiram exposure in the induction of lipid metabolism (18). The liver is an important metabolic and detoxifying organ in animals and humans, which is regarded as one of the main target organs for multiple stimulations including pesticides, heavy metals and various environmental pollutants (48–50). Therefore, pesticide residues in feed are likely to affect liver health, which will cause great damage to poultry production. However, study on thiram toxicities to liver of chicken is still lacking. In this study, we explored the effect of thiram exposure on liver metabolism in chickens.

In this study, 97 differential metabolites were totally recognized, which was closely related to multiple metabolic pathways including glycerophospholipid metabolism, porphyrin and chlorophyll metabolism, primary bile acid biosynthesis, steroid hormone biosynthesis and glycosylphosphatidylinositol (GPI)-anchor biosynthesis. These metabolic pathways may play an important role in the hepatotoxicity induced by thiram. Remarkably, some of the decreased metabolites including ginsenosides, arbekacin, coproporphyrinogen III, Fusidic acid, marmesin and fluorouracil play important roles in antioxidant capacity, anti-cancer and oxygen transport. Ginsenosides were widely recognized because of multiple beneficial effects, such as inhibiting the growth of cancer cells, inducing tumor cell apoptosis, reversing the abnormal differentiation of tumor cells, and anti-tumor metastasis (51). Moreover, ginsenoside has been demonstrated to improve immunity and antioxidant capacity of host (52). Zhang et al. revealed that the concentrations of aspartate aminotransferase and alanine aminotransferase in thiram-induced chickens significantly increased, but antioxidant enzyme dramatically decreased, suggesting liver injury and antioxidant dysfunction (53). Therefore, we speculated that the decreased ginsenoside may be one of the important pathways for thiram exerts its toxic effects and cause antioxidant dysfunction. Previous research indicated that arbekacin have an inhibitory effect on multiple pathogens such as *Pseudomonas aeruginosa, Klebsiella pneumonia* and *Acinetobacter baumannii* (54). Moreover, arbekacin can be used for treating multiple drug resistant pneumonia and septicemia as well as infections caused by resistant *Staphylococcus Aureus* (55, 56). Coproporphyrinogen III play a key role in the production of heme (57, 58). Heme is also an important component of hemoglobin, which plays a key role in the transport of oxygen. Oxygen has been demonstrated to play key roles in blood vessel development and bone formation (59). Previous studies indicated that the chickens exposed to thiram showed weight loss, accompanied by angiogenesis disorder and tibial dyschondroplasia (60, 61). Therefore, decreased coproporphyrinogen III may be one of the causes of angiogenesis disorder and abnormal bone development of chickens.

Fusidic acid can treat infections induced by methicillin-susceptible and methicillin-resistant Staphylococcus aureus (62). Marmesinpossess multiple pharmacological functions including anti-inflammatory, antihepatotoxic and antitumor activities (63, 64). Fluorouracil has anti-cancer effects (65). Moreover, we observed increased levels of L-palmitoylcarnitine, quinestrol, 7-ketocholesterol, and tetrabromodiphenyl ether during thiram exposure. L-palmitoylcarnitine is an ester derivative of carnitine, which participated in fatty acids metabolism and its abundance increased during hepatic lipid accumulation (66, 67). Consistent with this study, Sheng et al. indicated that the abundance of L-palmitoylcarnitine increased significantly in zebrafish exposed to organic pollutants (68). Moreover, increased L-palmitoylcarnitine was closely related to poorer prognosis in patients with chronic heart failure (69). Quinestrol can disrupt internal secretion and cause fertility disorders by inducing testicular damage (70). Moreover, quinestrol can increase the levels of serum MDA and aggravate the oxidative damage of cells (71). As a pro-oxidant and pro-inflammatory molecule, 7-ketocholesterol not only induces inflammation and nerve cell damage, but also affects membrane permeability and causes oxidative stress (72). Tetrabromodiphenyl ether is known to possess reproductive toxicity, which weaken sperm activity and increase the quantity of abnormal sperm (73). Moreover, tetrabromodiphenyl ether has also been demonstrated to induce liver inflammation and promote the expression of inflammatory genes including IL-6, TNF-α and IL-l β (74). Increasing evidence demonstrated that long-term pesticide exposure can result in cancer and reproductive disorders. In this study, we observed significant changes in metabolites associated with anti-cancer, oxidative stress and reproductive function, indicating that thiram may also be a potential cancer-inducing factor. Previous study indicated that thiram exposure could induce liver autophagy and apoptosis. Notably, some studies also showed that oxidative stress could cause the initiation and development of apoptosis and autophagy. Therefore, thiram induced liver apoptosis and autophagy may be mediated by differential metabolites related to oxidative stress.

In conclusion, this study investigated the effect of thiram exposure on liver metabolism in chickens. Results showed that thiram exposure can significantly alter liver metabolism, characterized by significant changes in some metabolites and metabolic pathways. These results filled in the blank of thiram exposure on liver metabolism characteristics of chickens, and conveyed an important message that hepatic metabolic disorder may be one of the important ways thiram affects broiler liver metabolism. Moreover, this study will help prevent and control the effects of thiram on liver metabolism in chickens from the perspective of liver metabolism.

## Data availability statement

The original contributions presented in the study are included in the article/supplementary material, further inquiries can be directed to the corresponding author.

## Ethics statement

The study was performed under the instructions and approval of Ethics Committee of the Wenzhou Vocational College of Science and Technology.

## Author contributions

MW and LW conceived, designed the experiments, and wrote the manuscript. DZ, HLu, HLi, ZL, XS, CW, and YZ contributed sample collection and reagents preparation. MW analyzed the data. SS and MS revised the manuscript. All authors reviewed the manuscript.
